# Hydrolysis of lignocellulose to succinic acid: a review of treatment methods and succinic acid applications

**DOI:** 10.1186/s13068-022-02244-5

**Published:** 2023-01-02

**Authors:** Shuzhen Zhou, Miaomiao Zhang, Linying Zhu, Xiaoling Zhao, Junying Chen, Wei Chen, Chun Chang

**Affiliations:** 1 College of Chemical Engineering, Zhengzhou University, Zhengzhou, China; 2 Henan Key Laboratory of Green Manufacturing of Biobased Chemicals, Puyang, China; 3 College of Management Engineering, Zhengzhou University, Zhengzhou, China; 4 State Key Laboratory of Motor Vehicle Biofuel Technology, Nanyang, China; 5 Henan Center for Outstanding Overseas Scientists, Zhengzhou, China

**Keywords:** Lignocellulose, Biological enzyme, Hydrolysis, Glucose, Succinic acid

## Abstract

Succinic acid (SA) is an intermediate product of the tricarboxylic acid cycle (TCA) and is one of the most significant platform chemicals for the production of various derivatives with high added value. Due to the depletion of fossil raw materials and the demand for eco-friendly energy sources, SA biosynthesis from renewable energy sources is gaining attention for its environmental friendliness. This review comprehensively analyzes strategies for the bioconversion of lignocellulose to SA based on the lignocellulose pretreatment processes and cellulose hydrolysis and fermentation principles and highlights the research progress on acid production and SA utilization under different microbial culture conditions. In addition, the fermentation efficiency of different microbial strains for the production of SA and the main challenges were analyzed. The future application directions of SA derivatives were pointed out. It is expected that this research will provide a reference for the optimization of SA production from lignocellulose.

## Introduction

**Fig. 1:**
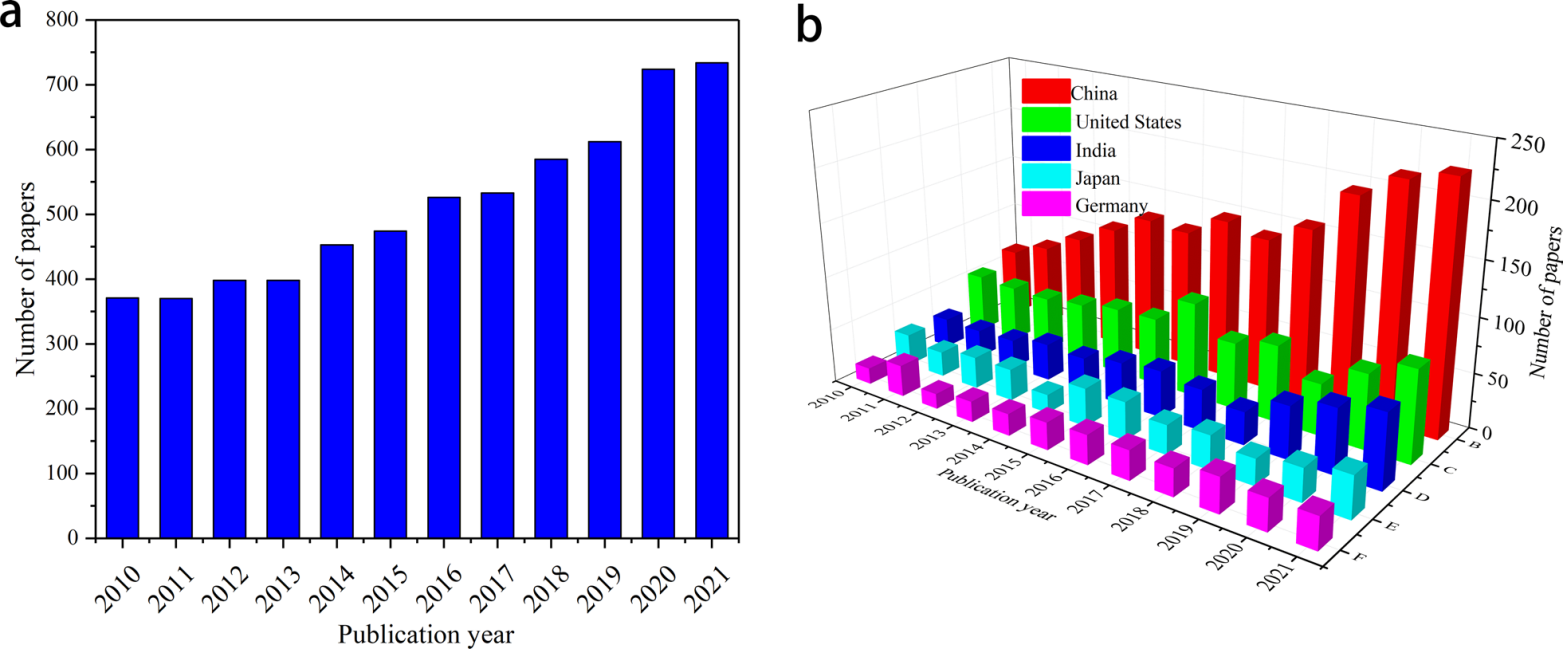
**a** The number of papers on succinic acid-associated research from 2010 to 2021. **b** The number of papers on succinic acid-associated research trends in different countries

The past decade has seen an exponentially increased interest in the production of special chemicals from bio-based resources [1] because of the severe environmental degradation caused by the use of fossil fuels in the chemical industry. The application of biomass raw materials for succinic acid (SA) production through fermentation can reduce costs and the chemical dependence on existing fossil-based reserves and contribute to the realization of the production target of SA-based consumer products [2]. Succinic acid, as a dicarboxylic acid with a C_4_ structure, is the starting material for producing various fine chemicals, food additives, biodegradable plastics, surfactants, and chemicals [3, 4]. Its extensive application has enabled it to become a promising basic chemical substance [5]. Succinic acid has been identified by the United States Department of Energy (DOE) as one of the 12 value-added bio-based platform chemicals [6]. The market potential of SA and its direct derivatives is estimated to be as high as 245,000 tons every year, while the market size of SA polymers is estimated to be 25 million tons every year [7]. This paper explored the research trends of SA from 2010 to 2021 in the Web of Science database by bibliometric analysis [8]. The retrieval results are shown in Fig. 1. As can be seen from Fig. 1a, in the past 11 years, the research on SA generally showed an increasing trend, and the number of publications in 2021 was about twice that in 2010. According to the analysis of Fig. 1b, five countries (i.e., China, the United States, India, Japan, and Germany) paid more and more attention to SA. China surpassed the United States to boast the most published papers related to SA worldwide. Bibliometric analysis indicated the dramatic increase in the number of papers in China in the past three years. Increasing attention has been paid to the impact of SA utilization. In conventional chemical synthesis, maleic anhydride from petrochemical feedstock is the key substrate for SA, and Pd/BN catalysts have been used for the preparation of succinic acid by hydrogenation of maleic anhydride [9]. Despite the high conversion rate, many problems remain to be solved, such as the complex operations, high energy consumption and harsh reaction conditions. Therefore, much attention has been paid to the biosynthesis of SA and a lot of relevant studies have been conducted [10]. Compared to chemical processing, biosynthesis has a wider range of raw materials and lower costs [11].

The International Energy Agency (IEA) predicts that fossil feedstocks will decline significantly by about 75% by 2035. The depletion of fossil feedstocks and the strong consumer demand for eco-friendly energy have led governments and the chemical industry to shift to biosynthesis for a sustainable global economy [12]. Lignocellulose is a potential candidate to replace fossil resources for the synthesis of chemicals, materials and fuels and is the most abundant biomass with an annual production of 220 billion tons [13]. At present, lignocellulose is used to produce SA by microbial fermentation, which contributes to the reduction of carbon dioxide emissions [14]. However, the cost-effectiveness of SA production from lignocellulose is closely related to the yield of released sugars during pretreatment and/or subsequent enzymatic hydrolysis. Moreover, the traditional synthesis methods of SA are plagued by high production costs, environmental pollution, etc. In order to cut the production cost of SA and enhance its market competitiveness, green microbial fermentation routes using low-cost carbon sources have become the research focus [15]. The development and utilization of lignocellulosic biomass, the most abundant renewable resource on earth, can reduce the environmental pollution of fossil energy and alleviate non-renewable resource shortages.

**Fig. 2:**
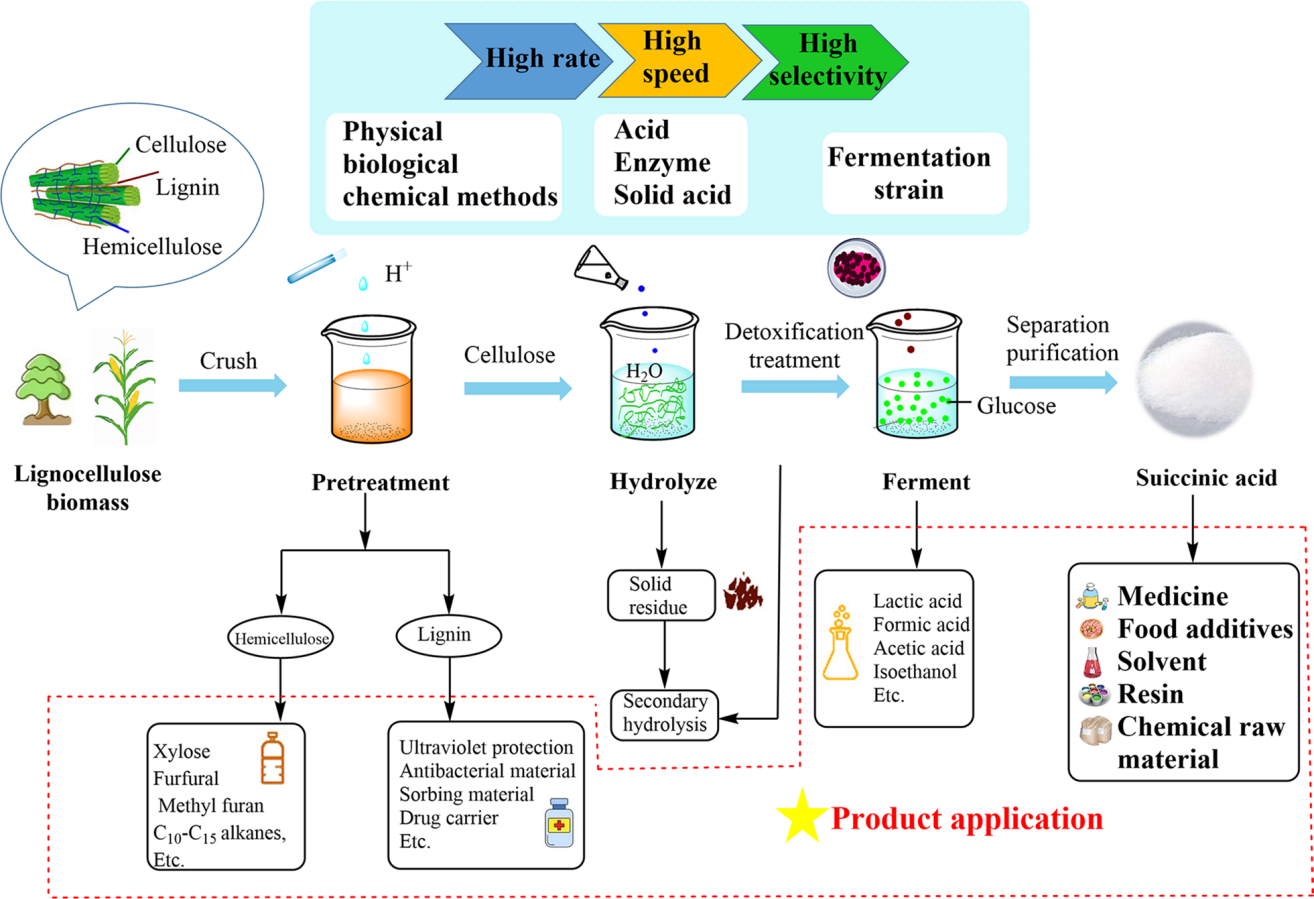
Flowchart of high-value utilization of lignocellulose

It is of great significance to prepare SA by the hydrolysis and fermentation of biomass resources containing numerous lignocellulose. The procedures mainly include biomass pretreatment, hydrolysis, saccharification of cellulose and catalytic conversion of glucose (Fig. 2). Firstly, due to the inherent complex polymer structure, highly ordered hydrogen bonds, and the indigestible nature of lignin that limits the conversion of lignocellulose [16, 17], the necessary pretreatment of lignocellulosic biomass was conducted [18]. Secondly, polysaccharides were hydrolyzed into monosaccharides, mainly by acid hydrolysis, enzymatic hydrolysis or solid acid-catalyzed hydrolysis. In the process of enzymolysis, the enzyme activity and hydrolysis efficiency can be improved by adding surfactants and other substances. The hydrolysis was followed by chemical or biological transformation into C_2_–C_6_ structural blocks for further synthesis of chemical products [19]. In the end, SA was produced by the microbial fermentation of sugar. Under optimized conditions, the yields of 134.25 g/L at the laboratory scale can be achieved [20]. However, production costs and feasibility should be considered during the biological production of SA on a large scale. New natural strains should be screened according to the metabolic strategy to further improve the yield of SA under industrial conditions. Currently, many studies focus on the regulation of metabolic pathways, genetic engineering techniques, and optimization of fermentation processes and culture media to improve SA production.

In this paper, technologies and methods of lignocellulose bioconversion into SA were comprehensively discussed from the aspects of lignocellulose pretreatment, cellulose hydrolysis, and glucose fermentation, with the emphasis on research progress of SA production under different microorganism co-culture conditions. Moreover, the applications and prospect of SA and its derivatives were summarized.

### Lignocellulosic pretreatment for succinic acid

**Fig. 3:**
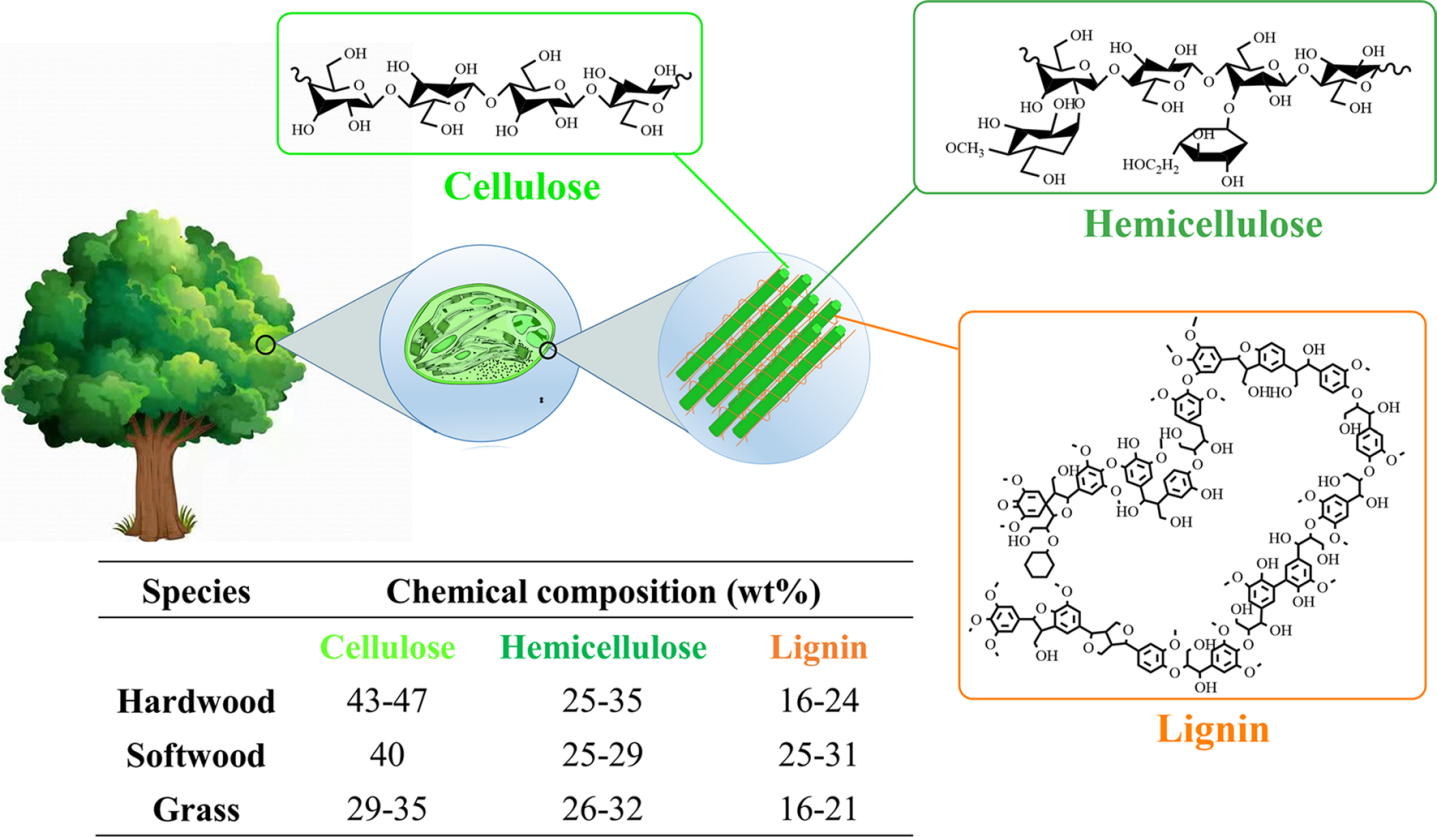
Schematic diagram of position, structure and content of cellulose, hemicellulose and lignin in lignocellulosic biomass [21]

One procedure to improve SA yield is the separation of cellulose from biomass prior to hydrolysis, indicating the significance of the pretreatment of lignocellulosic material to overcome the stiffness of material so as to make the sugar available for subsequent enzymatic and microbial fermentation. Generally, lignocellulosic biomass mainly comprises cellulose (29–47 wt%), hemicelluloses (25–35 wt%), and lignin (16–31 wt%) [21, 22]. The position, structure and content of cellulose, hemicellulose and lignin in lignocellulosic biomass are shown in Fig. 3. The recalcitrance of the crystalline cellulose in lignocellulose to enzymatic hydrolysis is mainly due to the strong intermolecular interactions, including hydrogen bonding and van der Waals force [23]. Hydrogen bonds enable the lignin to tightly wrap cellulose and hemicellulose, leading to the lack of porosity, the existence of side chains, the crystallization of cellulose, and the impermeability of natural lignocellulose with high molecular weight and heterogeneous matrix. Consequently, hydrogen bonds show high-stress resistance, which hinders the catalytic hydrolysis of cellulose [24]. One procedure to improve SA yield is the separation of cellulose from biomass prior to hydrolysis. In order to expose cellulose and improve its accessibility in lignocellulose for subsequent enzymatic and microbial fermentation, lignocellulose should be pretreated to remove hemicellulose and lignin. Effective pretreatment methods must meet the following requirements: (1) they can change the dense structure of natural lignocellulose, destroy the chemical and physical connection between cellulose, lignin, and hemicellulose, and remove soluble inorganic salts, lignin and hemicellulose; (2) they can reduce cellulose crystallinity, improve the surface accessibility of cellulose, and avoid degradation; (3) they can avoid the generation of by-products inhibiting hydrolysis fermentation, enable the selection of pretreated raw materials that can be directly hydrolyzed and fermented, and require reduced production cost; (4) they can increase the porosity of raw materials to promote the effective cellulase system–cellulose contact so as to improve the yields in subsequent saccharification and fermentation.

Currently, physical, chemical, biological, and combinatorial pretreatment methods are used. Physical pretreatment is the technology of changing the appearance and structure of lignocellulosic biomass by crushing, extrusion, steam explosion [25], ultrasonic treatment, microwave treatment, high-energy radiation treatment, and liquid hot water (LHW) [26]. Although these procedures are quick, time-saving, and environment-friendly, the costs are high [27]. Chemical pretreatment is the method of destroying the internal structure of biomass by adding acid, alkali, organic solvent, etc. Despite the simple operation and high efficiency, this method may cause secondary pollution and show certain limitations [28]. Compared with the other pretreatment methods, biological pretreatment consumes fewer chemical substances and less energy. Fungi (brown rot fungi, white rot fungi, and soft rot fungi) show excellent biodegradability in the process of lignin removal from wheat straw materials [29], making the biological pretreatment method environment-friendly. However, the long fermentation time and many by-products will increase the cost of fermentation, and more efficient biological strains need to be cultivated through strain improvement and fermentation medium optimization [30–32]. Additionally, different pretreatment methods show varying specificity for the change in the physical and chemical structure of crop straw [33]. Although a single pretreatment technology can improve the hydrolytic efficiency and the acidic substance yield, its large-scale application in China may be restricted [34]. Therefore, in recent years, combined pretreatment technology with synergistic effects has been developed and achieved large-scale application. Table 1 shows the advantages and disadvantages of different lignocellulose pretreatment methods. Physical pretreatment aims to reduce the particle size of lignocellulose, increase the surface area, and reduce the degree of polymerization and crystallinity of cellulose. However, lignin does not degrade during physical pretreatment, and therefore physical pretreatment is usually combined with other pretreatments. During chemical pretreatment, chemical reagents such as acids and bases are used to promote the delignification of lignin and expose cellulose and hemicellulose. Although chemical pretreatment is one of the most effective pretreatment technologies, the higher requirement for chemical reagents leads to higher capital investment and more environmental hazards. Biological pretreatment is currently the most cost-effective method, but the long pretreatment time needs to be further shortened. In general, a single pretreatment technology usually fails to achieve the expected pretreatment results [35].

**Table 1:** Pretreatment methods of lignocellulose

Pretreatment types	Pretreatment methods	Characteristics	Pretreatment effect yield	References
Physical pretreatment	Ultrasonic pretreatment, steam pretreatment, explosion pretreatment, etc.	Simple principle; convenient operation, high energy consumption, and high cost	Succinic acid (45.7 kg/100 t) Glucose (89.55%)	[98] [99]
Chemical pretreatment	Acid pretreatment, alkaline pretreatment, Ionic liquid pretreatment, etc.	Simple operation; high efficiency; potential secondary pollution	Succinic acid (71%) Succinic acid (63.8%) Succinic acid (57.1%)	[100] [101] [102]
Biological pretreatment	Germ pretreatment, fungus pretreatment, enzyme pretreatment, etc.	Time-consuming; pollution-free; mild conditions; low energy consumption	Lignin removals (38.7%) Free carbon (97%) Enzyme activity (5.6–24.0 U/g)	[103] [104] [105]
Combined pretreatment	Physicochemical pretreatment, biochemical pretreatment, etc.	High efficiency; low cost and energy consumption; environment- friendly	Succinic acid (79.0%) Succinic acid (83.0%) Succinic acid (19.3 g/L)	[42] [106] [107]

The combined pretreatments [36–38] have better performance and can be used for the conversion from lignocellulose to SA. Thermal pretreatment (alkali heat) in the optimal range of 75–125 °C can dissolve lignin in the presence of alkali (sodium hydroxide, sodium carbonate, alkaline peroxide, etc.), reduce the crystallinity of lignocellulose by swelling, and thus expand the specific surface area of cellulose [39]. To date, the maximum lignin and hemicellulose removal rates from corn stover using NaOH thermal pretreatment reached 54.09% and 67.67%, respectively, while the relative content of cellulose increased to 51.65% [38, 40]. Zhang et al. [41] further demonstrated that reducing sugar yield could contribute to a high level of corn stover (23.07 g/100 g) after NaOH thermal pretreatment and enzymatic hydrolysis, indicating that this pretreatment method has good cellulose retention properties. Xi et al. [42] pretreated sugarcane bagasse by ultrasonic combined with dilute acid, and the yield of SA increased to 23.7 g/L with a SA yield rate of 79.0% and a productivity of 0.99 g/L/h.

Although combined pretreatment has higher SA production efficiency than single pretreatment, the high costs and complex processes hinder its application. Therefore, the efficient utilization of lignocellulose remains difficult due to its recalcitrant structure. In practical engineering applications, it is of great significance to compare the superiority and drawbacks of different pretreatment methods according to raw materials; it is also indispensable to select the ideal method to optimize the biomass utilization rate and the efficiency of fermentation and acid production, reduce the organic matter loss, and avoid the incorporation of anaerobic inhibitors. Moreover, both economic effectiveness and environmental safety should be taken into account to ensure low cost, low energy consumption, and environmental friendliness of the process.

### Hydrolysis and saccharification of cellulose

In the SA production from lignocellulose raw materials, hydrolysis and saccharification of cellulose are the first steps and the rate-limiting step. The common hydrolysis methods are acid hydrolysis, enzymolysis, and solid acid-catalyzed hydrolysis. Efficient cellulose hydrolysis by different methods is beneficial to improve the utilization efficiency of lignocellulosic biomass [43], thus increasing the SA yield. Cellulose is a long-chain polysaccharide compound with a high crystallization area and is connected by d-glucose and β-1,4 glycosidic bonds. Through large numbers of intra- and inter-chain hydrogen bonds, cellulose chains assemble into regularly arranged crystals and pores filled with irregular chains (amorphous regions) [44]. It is challenging for cellulose to achieve self-degradation and produce glucose, and hydrolysis only occurs in the presence of catalysts. Under mild conditions, the initial stage of cellulose hydrolysis is a heterogeneous reaction. Homogeneous proton catalysts (such as H_2_SO_4_ and HCl) are usually effective because they can penetrate the heterogeneous cellulose matrix. The hydrolysis technology of cellulose is mainly divided into acid hydrolysis and enzyme hydrolysis. The key to producing downstream products from cellulose is the hydrolysis of cellulose into glucose, i.e., cellulose saccharification.

#### Acid hydrolysis

**Fig. 4:**
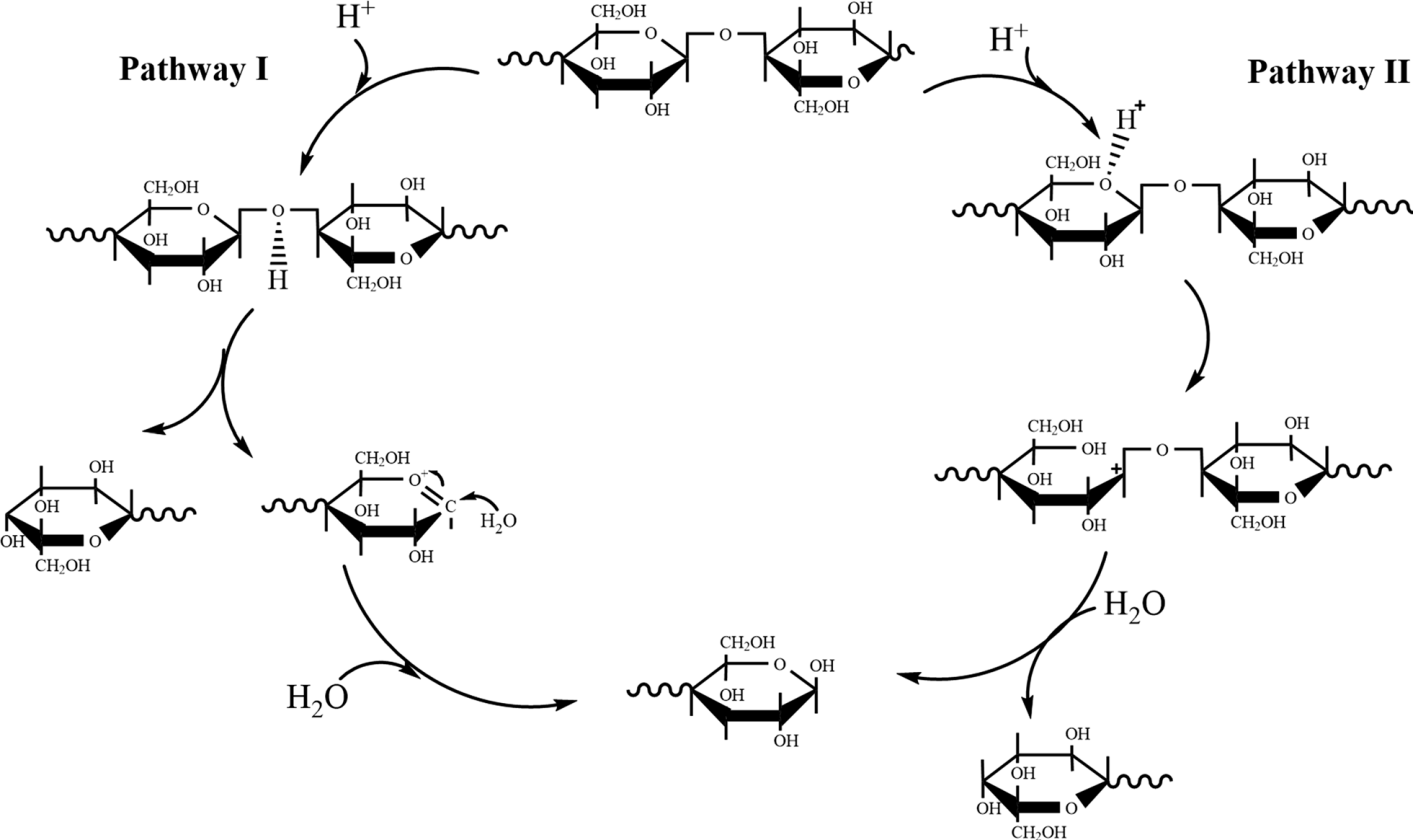
Mechanism of acid-catalyzed hydrolysis and saccharification of cellulose [125]

The hydrolysis of cellulose to glucose is the first step in the effective utilization of cellulose [45], where liquid catalysts mainly include inorganic acids (such as hydrochloric acid, sulfuric acid and phosphoric acid) and organic acids (such as oxalic acid, aryl sulfonic acid and formic acid). Generally, the hydrolysis and saccharification of cellulose activate the oxygen atoms on the glycosidic bond connecting the glucose units and break the C–O bond to obtain glucose [46]. Among them, the mechanism of acid hydrolysis is to catalyze cellulose to produce glucose (Fig. 4), mainly involving three parts: (1) H^+^ ionized in acid solution attacks and rapidly protonates the oxygen atom on the glycosidic bond (pathway I); (2) the C–O bond breaks, and the positive charge on the glycosidic bond is transferred to the C atom of the glucose unit, forming the carbocation C^+^; (3) water molecules attack carbocation C^+^ to obtain free residue glucose and form hydronium ion H_3_O^+^ [47]. In addition to this pathway, the oxygen atom in the six-membered sugar ring can undergo the above reaction via acid catalysis (pathway II) according to the chemical structure analysis. However, research on disaccharides suggested that hydrolysis mainly followed pathway I. In contrast, pathway II was only a possible reaction path, and its influence and function on cellulose hydrolysis remain unclear [48].

Liquid can penetrate inside the cellulose particles and increase the contact with glycosidic bonds and hydrolysis rates, which helps to improve the conversion rate. Cellulose acid hydrolysis is simple and has a short production cycle. Moreover, the high temperature and low pH during the acid-catalyzed hydrolysis may help to eliminate bacterial contamination in large-scale continuous fermentation. Diaz-Blanco et al. [49] studied the pretreatment of dilute sulfuric acid with a pretreatment temperature of 160–200 °C and an acid concentration of 0.005–0.015 g/mL. The results showed that the optimum pretreatment conditions for agave acid were 180 °C and 0.0124 g/mL sulfuric acid at 10% biomass loading, under which the recovery rate of hemicellulose sugar and glucose reached 87% and 68%, respectively. Martin et al. [50] investigated the effects of dilute sulfuric acid pretreatment on glucan recovery, enzyme conversion, and by-product formation. The results showed that at a pretreatment temperature of 195 °C, a sulfuric acid concentration of 0.6%, and a pretreatment time of 50 min, the maximum enzymatic conversion rate of pretreated cellulose was 83.8%, and the maximum conversion rate of glucan to glucose was 72%.

However, the excessive acidity of the liquid acid catalyst easily leads to the continuous catalysis of sugar products and side reactions, which causes decreased hydrolysis selectivity. Therefore, the mechanism of controlling and achieving efficient hydrolysis of cellulose to sugar using liquid acids has become the focus of research.

#### Enzymatic hydrolysis

Compared with acid hydrolysis, enzymatic hydrolysis of cellulose features mild technological conditions and no pollution. Cellulose hydrolysis by cellulase is similar to its heterogeneous dilute acid hydrolysis (hydrochloric acid or sulfuric acid). Both of them break the β-1,4-glycosidic bond of cellulose macromolecules under the catalysis of cellulase and H^+^, and cellulose hydrolysis produces glucose, cellobiose and oligosaccharide [51]. However, similar mechanisms of enzymatic hydrolysis and acid hydrolysis may not produce similar effects. This is because enzyme molecules and hydrochloric acid/sulfuric acid molecules (as proteins) are greatly different in structure and properties, and the complexity of fiber structure makes the two hydrolyses have different effects on fiber structure and properties.

**Fig. 5:**
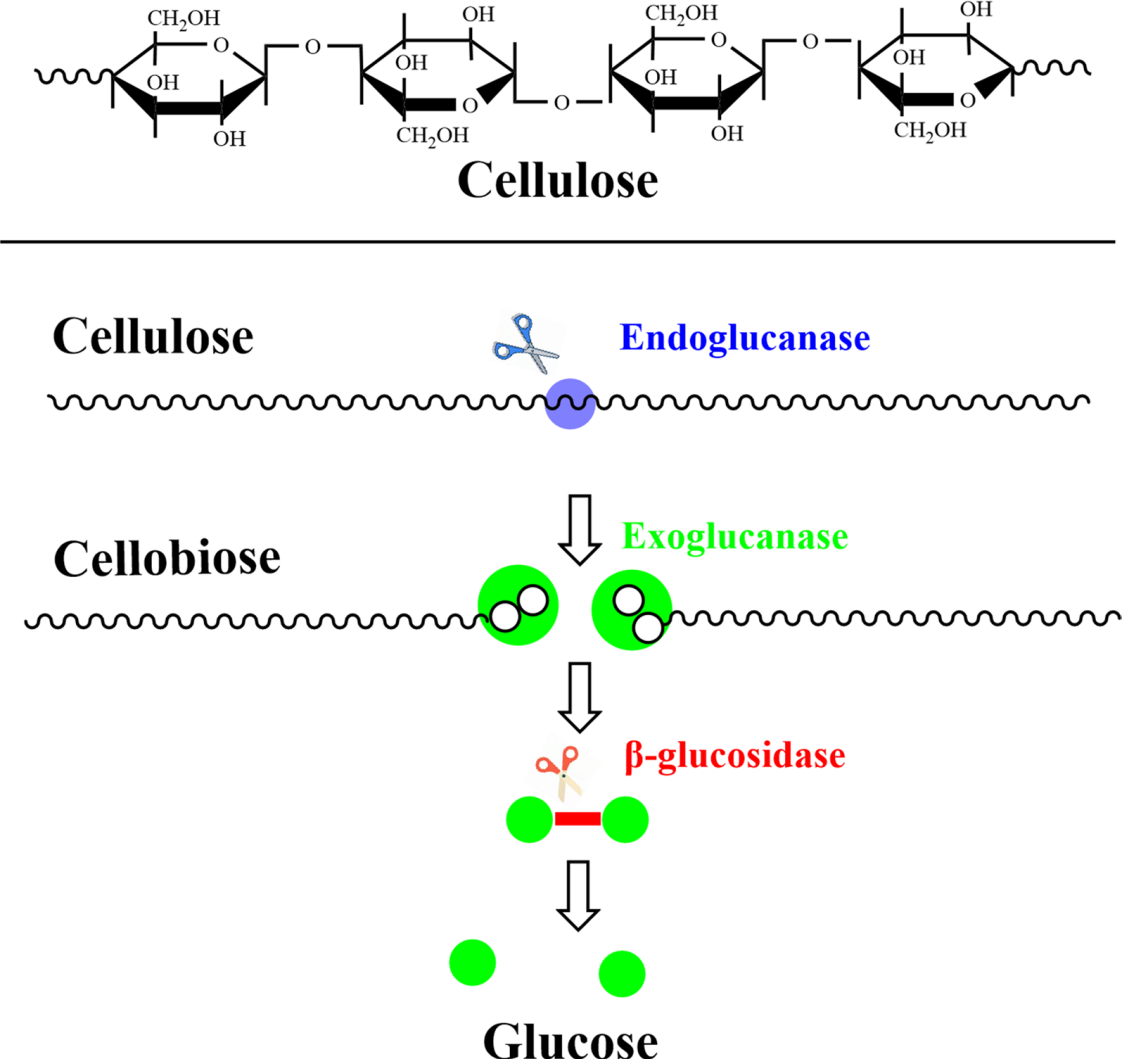
Mechanism of cellulase hydrolysis [126]

Cellulase is a complex enzyme comprising endoglucanase, exoglucanase, and β-glucosidase [52]. Compared with acid hydrolysis of cellulose, enzymatic hydrolysis of cellulose is more widely used owing to its mild conditions, strong specificity, easy control, and few by-products. The process mechanism of enzymatic hydrolysis of cellulose is shown in Fig. 5. As can be seen, enzymatic hydrolysis occurs under the combined action of exoglucanase and endoglucanase on cellulose. Specifically, endoglucanase acts on the β-1,4-glycosidic bond in the amorphous region of the cellulose molecular structure, breaking it to shorten the chain length structure of cellulose while producing numerous reducing and non-reducing ends. Subsequently, exoglucanase acts on the non-reducing and reducing ends to generate cellobiose molecules [43]. Finally, cellulose is degraded into soluble oligoglucose under the synergistic action of the two enzymes, and then single-molecule glucose can be obtained under the action of β-glucosidase [53].

The addition of some additives, such as proteins, surfactants, and polymers, to the enzymatic hydrolysis system can increase the sugar yield and reduce the dosage of enzymes. Among them, the addition of protein can improve the accessibility of cellulase to cellulose, i.e., increasing the probability of the cellulose–substrate reaction. Some proteins can also reduce cellulase inactivation by improving the stability of cellulase. Moreover, growing attention has been paid to improving cellulase hydrolysis by adding surfactants. Surfactants can help cellulase to hydrolyze lignocellulose, enhance the yield of glucose and xylose and the removal rate of lignin, and reduce the non-reactive adsorption of cellulase and lignin. In addition, surfactants influence the activity of cellulase and can improve the activity and stability of enzymes. The effects of cationic surfactants with varying alkyl chain lengths on the enzymatic hydrolysis of microcrystalline cellulose and the surface charge of cellulase were studied. The results showed that the enzymatic hydrolysis rate of microcrystalline cellulose increased linearly from 42.1% to 61.4% after adding surfactants. Cetyltrimethylammonium bromide C (16) TAB increased the enzymatic hydrolytic efficiency of corncob with high solid content from 35.0% to 56.3% and reduced the cellulase dosage by about 60% to obtain the same glucose yield. The study lays a foundation for recycling cellulase using surfactants and provides a design direction for obtaining cellulase with higher activity and better stability by adjusting its hydrophilicity and charge [54]. At low concentrations, surfactants can increase the flexibility of enzyme molecules, which facilitates the interaction and combination of molecules with the substrate, thus increasing the activity of the enzyme. At low concentrations of cationic surfactants, the surface layer of cationic cellulase changes the conformation of the enzyme, which may promote electron transfer in the enzyme molecule and thus enhance the activity of cellulase [55]. Tween is polysorbate with good hydrophilicity due to many polyoxyethylene groups in its molecule. Tween surfactant adsorbs on the lignin surface, mainly affecting the substrate and enzyme to improve the enzymatic hydrolytic efficiency of lignocellulose. Its hydrophilic group exerts a steric hindrance effect on cellulase to prevent the hydrophobic part of lignin from interacting with cellulase.

Tween 80 can improve the adsorption and desorption performance of cellulose [56]. It can reduce the ineffective adsorption of endoglucanase and allow free endoglucanase to produce more free chain end-groups. It promotes cellobiohydrolase to remove cellobiose units from free chain end-groups on cellulose and degrade cellulose. Moreover, it can improve the activities of cellobiose and endoglycosidase in cellulase and promote the synergy between the two enzymes and exoglycosidase [57, 58]. Tween 80 surfactant is used to improve enzymatic hydrolysis. Under optimized conditions (550 mL of CSF pulp freeness, 28 FPU/g of enzyme dosage, and 1.66% of Tween 80), the reducing sugar yield per unit of dry regenerated substance was increased by eightfold compared with that of untreated substance, indicating the facilitation of Tween 80 for lignocellulose hydrolysis to SA [59].

#### Hydrolysis of cellulose catalyzed by solid acid

Homogeneous acid and cellulase are the most common catalysts for the hydrolysis of cellulose to glucose. However, liquid catalysts such as sulfuric acid are costly and low in efficiency, and the glucose generated during acidolysis is easily degraded, resulting in unrecoverable sulfuric acid waste [60]. In addition, the enzymatic reaction time is long. Aiming to overcome the above shortcomings, increasing solid acid catalysts have been employed to hydrolyze cellulose to prepare glucose in recent years [61]. From the perspective of green chemistry and industrialization, solid acid catalysts, such as acid resin, metal oxide, H-zeolite, heteropoly acid, functionalized silica, supported metal, immobilized IL, carbonaceous acid, and magnetic acid, are separable, recyclable, and reusable and show excellent catalytic activity for cellulose hydrolysis to glucose [62].

**Fig. 6:**
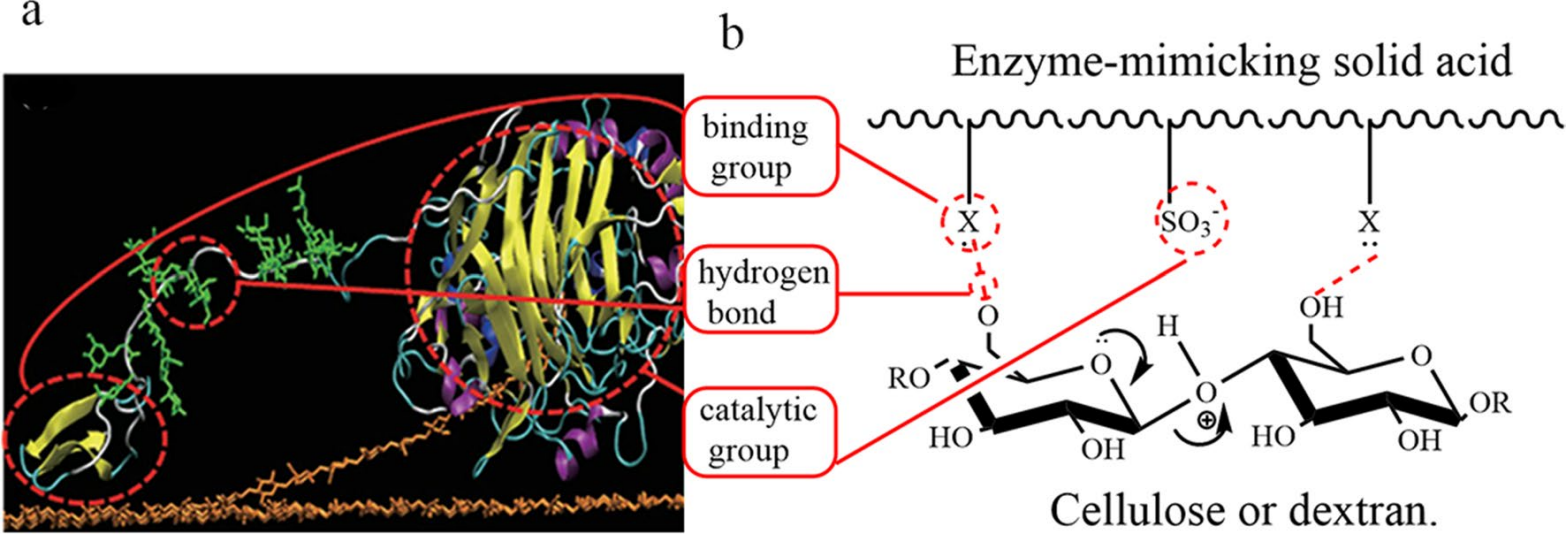
Mechanism of enzymatic hydrolysis of cellulose or dextran by simulated cellulase with enzyme-like solid acid [127]

Unlike liquid acids, solid acids and cellulose are insoluble in conventional solvents, and a severe mass transfer restriction exists between them [63], resulting in the far lower catalytic performance of solid acids than that of inorganic acids and other homogeneous liquid acids. It has been found that [64] cellulase can hydrolyze cellulose mainly due to its binding groups and catalytic groups (Fig. 6a); the binding group is mainly responsible for the formation of hydrogen bonds between cellulase and hydroxyl groups on cellulose chains, thus narrowing the cellulose–cellulose distance; in contrast, the catalytic group breaks the β-1,4 glycosidic bond of the cellulose chain; the synergistic effect of the binding group and the catalytic group significantly improves the hydrolytic efficiency of cellulase. Therefore, by simulating the hydrolysis of cellulose by cellulase, group X with an affinity for cellulose, such as –C, –COOH, –OH, and –B (OH), is introduced into solid acid catalysts (Fig. 6b). These groups can form hydrogen bonds with cellulose, break the intramolecular and intermolecular hydrogen bonds of cellulose, and synergize with catalytic groups –SO_3_H or –COOH on solid acids to improve the hydrolytic efficiency of cellulose.

Compared with traditional methods, solid acid-catalyzed cellulose hydrolysis is green, pollution-free, non-corrosive, and easy to recover [65], but the low hydrolytic efficiency hinders its popularization and application. Onda et al. [66] prepared an activated carbon-based solid acid catalyst, and the glucose yield was 40.5% in a 423 K aqueous solution after 24 h reaction. J. Pang et al. [67] prepared CMK-3 catalysts with sulfonated activated carbon at 523 K, and the glucose yield was as high as 74.5%. The more contact between the solid acid catalyst and cellulose substrate, the easier it was for the β-1,4 glycosidic bond to be broken [68]. Due to choline substituents, its excellent adsorption capacity greatly promotes cellulose to enter the catalytic site. A magnetic carbon-based solid acid catalyst derived from sucralose with cellulose binding and catalytic sites has been prepared by incomplete carbonization of sulfonated sucralose and Fe^3+^ at high temperatures and loading-Fe_2_O_3_ nanoparticles on the catalyst surface. At 363 K, the glucose yield of microcrystalline cellulose pretreated by ball milling reached up to 32%. The catalyst maintained 90% of activity after five cycles. Magnetic properties were introduced into the catalyst system, which was a combination of the great catalytic ability of cellulose hydrolysis with the convenience of magnetic material separation [69].

Since the hydrolysis of cellulose and solid acids is a solid–solid reaction, the effective contact between the catalyst and the substrate limits the reaction. To ensure more effective cellulose hydrolysis using solid acids, the ball milling of cellulose or microwave heating was used to improve the contact and mass transfer between the two [70]. Additionally, the development of new solid acid catalysts by modifying solid acids, introducing groups, and other measures could render microscopic interaction of the catalysts with cellulose surface, thus improving the specific surface area [71] and thermal stability [72]. In this way, it is expected to improve the catalyst activity and increase the service life and application range of the catalyst. Furthermore, solid acid catalysts with better catalytic performance, more reasonable prices, and greener production can be developed through technological innovation and the introduction of frontier disciplines.

### Glucose fermentation to produce succinic acid

#### The main pathway of succinic acid biosynthesis

**Fig. 7:**
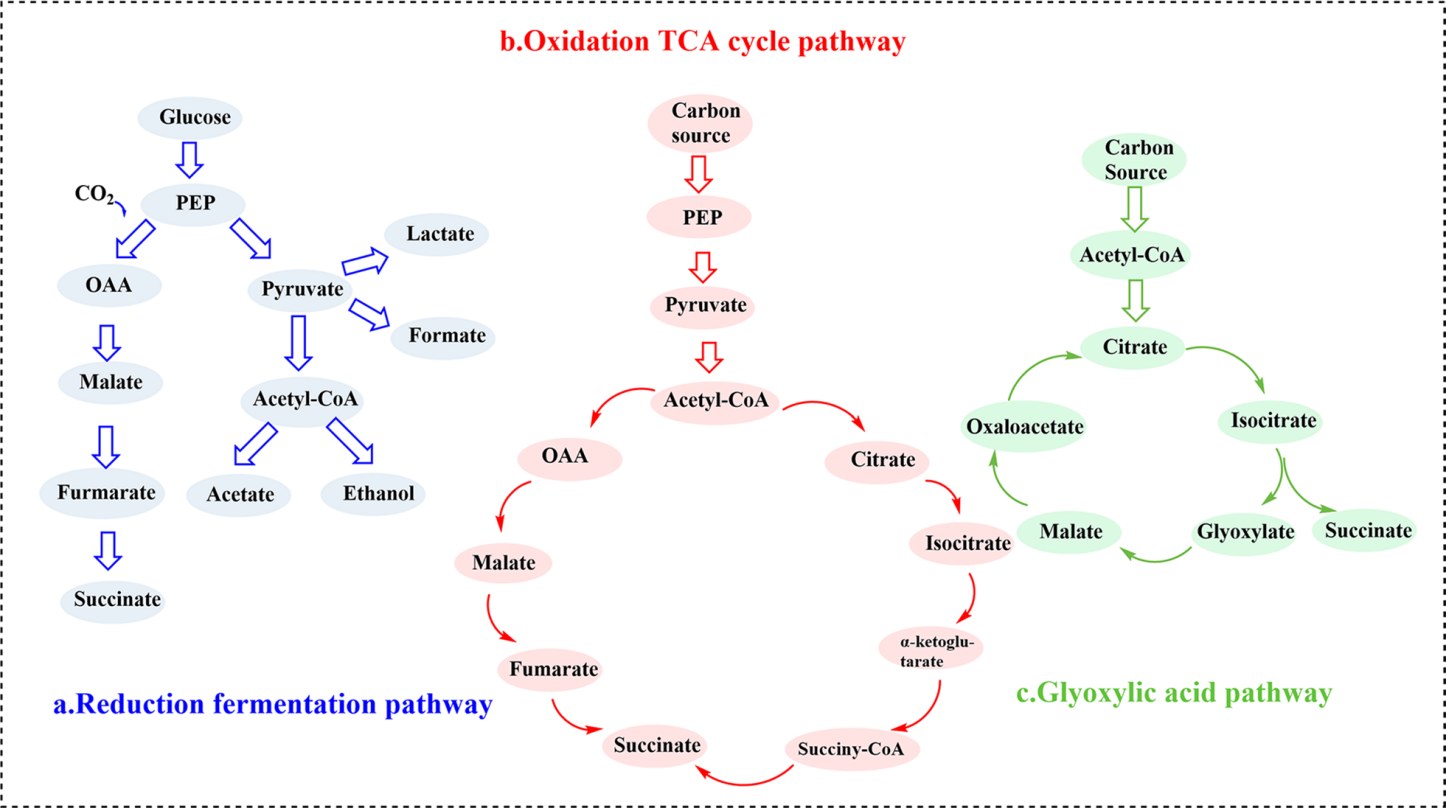
Three main metabolic pathways of succinic acid production [5]

Microbial fermentation is a method of producing SA and its derivatives from renewable and inexpensive resources by fermentation using bacteria or microorganisms [73]. Additionally, SA can be biosynthesized from sugars and used as a platform chemical for various chemical and polymer applications. Three major metabolic pathways are accessible to produce SA (Fig. 7): the reductive fermentation pathway, the glyoxylic acid pathway, and the TCA oxidation cycle pathway. (a) In the reductive fermentation pathway, also known as the reductive branch of the TCA cycle, phosphoenolpyruvate is converted into oxaloacetic acid, malic acid, and fumaric acid (FA) by a series of enzymes (phosphoenolpyruvate carboxykinase, malic dehydrogenase, fumarase, and fumarase), and forming SA eventually. Moreover, the formation of succinate consumes CO_2_, and CO_2_ fixation is crucial to the environment. However, by-products such as acetate, formate, lactate, and ethanol can also be produced. To concentrate on the metabolic flux of SA, the formation of by-products should be prevented. (b) The TCA oxidation cycle occurs in the mitochondria of eukaryotes. In the process, acetyl-CoA is converted into citric acid, isocitrate, ketoglutarate, succinyl-CoA, SA, FA, malic acid, and oxaloacetic acid, and then converted into citric acid to complete the cycle [74]. (c) The glyoxylic acid pathway is another pathway for SA production by some microorganisms, which supplements the TCA cycle intermediates. Acetyl-CoA from a carbon source, together with glyoxylic acid, is converted to SA under aerobic conditions suitable for acetate growth [75].

#### Metabolic strains producing succinic acid

Traditionally, SA is produced by the petrochemical route, although it can also be prepared by the hydrogenation of maleic acid/anhydride in the presence of Pd–Al_2_O_3_ catalyst or under the oxidation of 1,4-butanediol with oxygen in an alkaline solution in the presence of PD–C. However, these processes require precious metal catalysts, such as palladium, and cause environmental problems induced by fossil fuels. To address the above problems, metabolic engineering bacteria [76] were used to produce SA from a glucose carbon source through biochemical processes, during which glucose was used as a substrate, and the TCA cycle was adopted. Succinic acid-producing strains can be divided into three categories: (1) natural SA-producing strains: *Actinobacillus* succinogenes, *Anaerobiospirillum succiniciproducens*, and *Mannheimia succiniciproducens*; (2) prokaryotic genetic engineering strains: *Escherichia coli* (*E. coli*) and *Corynebacterium glutamicum*; (3) eukaryotic genetic engineering strains: *Saccharomyces cerevisiae* and *Yersinia lipolytica* [77]. Table 2 shows that the yield of SA has been very high in recent years and even exceeds 130 g/L at certain time points, but SA biosynthesis is still a hot research topic, mainly because production costs and feasibility should be considered during the large-scale biological production of SA. Although the current yield of SA at the experimental scale is high, it is still difficult to culture microorganisms and maintain an anaerobic environment at a commercial scale, which will cause additional costs. The SA production efficiency of biological routes needs further improvement, which can be achieved by metabolic pathway modulation, fermentation process optimization, culture media optimization and genetic engineering techniques.

**Table 2:** Succinic acid-producing strains with glucose as carbon source

Bacterial strain	Fermentation mode	Concentration (g/L)	Yield (g/g)	Productivity (g/L/h)	References
zATCC 55618	Naerobic	17.5	0.61	0.49	[15]
*Corynebacterium glutamicum*	Fed-batch	134.25	0.86	21.3	[20]
*Escherichia coli*	Anaerobic	133.45	0.89	2.97	[31]
NJ113	Fed-batch	56.2	–	1.00	[108]
*Actinobacillus succinogenes*	Repeated-batch	98.7	0.89	2.77	[109]
SD121	Fed-batch	116.2	1.13	1.55	[110]
130Z	Fed-batch	42.8	0.74	1.27	[111]
Δ*ldhA*/pXMJ19*pyc*	Anaerobic–aerobic	107	0.88	1.12	[112]
Tang1527	Dual phase, batch	89.4	1.24	1.27	[113]
AS1600a	Anaerobic, batch	84.26	0.88	0.96	[114]
PALFK	Fed-batch	78.4	1.64	6.02	[115]
*Succinogenes*	Batch	22.1	–	0.46	[116]
*Succinogenes*	Anaerobic	27.8	0.61	–	[10]
*Actinobacillus succinogenes*	Fed-batch	40.2	0.67	0.79	[117]
*Actinobacillus succinogenes*	Fed-batch	30	0.8	0.62	[118]
*Actinobacillus succinogenes*	Fed-batch	69.1	0.39	1.26	[119]
*Succinogenes* 130Z	Microbial cocultivation	32.5	0.39	0.14	[120]
succinogenes	Anaerobic	22.1	0.74	–	[121]
*Actinobacillus succinogenes*	Packed-bed biofilm reactor	43	0.58	22	[122]
KMG111	Batch	32.41	0.86	2.15	[123]
ATCC55618	Batch	26.7	0.621	3.33	[124]

Succinate accumulation in model strains can be enhanced by regulating key enzymes of the succinate metabolic pathway. *E. coli* is one of the most studied systems for SA production [47, 78, 79]. Under aerobic conditions, succinate is a metabolite of the *E. coli* TCA cycle, and in order for the metabolic flow to the TCA cycle reduction branch to produce succinate, several key carboxylases can be expressed. Among them, PEP carboxylase (PPC) and PEP carboxykinase (PCK) are the key enzymes that catalyze the reaction of phosphoenolpyruvate (PEP) to oxaloacetate (OOA) [80, 81]. PCK is mainly involved in the sugar xenobiotic pathway in *E. coli*, but it is a key enzyme in the carboxylation of PEP in SA-producing actinobacteria. PCK can produce one more molecule of ATP than PPC when involved in carboxylation; from the energy conservation aspect, PCK is more beneficial for SA production in the strain [82].

Genetic engineering techniques can improve strains at the genetic level to achieve high yields of SA [12]. Klasson et al. [47] studied the sucrose acid hydrolysis and fermentation yield in industrial sweet sorghum syrup by optimizing the transgenic SA-producing *E. coli* AFP184 strain. The results showed that the final concentration of SA was merely 27 g/L, but when grown on pure glucose, AFP184 consumed all fructose, proving its capability of producing a higher level of SA. This fully confirmed the great possibility of the genetically modified *E. coli* AFP184 in SA fermentation, including the sucrose expression. Aponte et al. [83] used brewer’s yeast to achieve higher SA concentrations in winemaking. Increasing phosphoenolpyruvate for SA synthesis by constructing a phosphotransferase system (PTS)-deficient *Corynebacterium glutamicum* mutant resulted in a 32.4% increase in SA yield [84].

The yield degradation cost can be reduced by optimization of the fermentation process and media. Stylianou et al. [85] used the engineered lipolytic bacterium PSA02004 to produce SA from biomass hydrolysate. By adopting a two-stage pH adjustment strategy, the pH value gradually decreased by 6–5.5 within 30 h; the concentration of SA was 54.4 g/L, the yield was 0.44 g/g, and the productivity was 0.82 g/L/h. In addition, NaOH consumption was reduced by 43% compared to that during the fermentation with a constant pH value of 6, showing that pH adjustment was an effective strategy for the sustainable production of SA from biological wastes. Kim et al. [86] used the LHW method to extract water-soluble hemicellulose from *Quercus mongolica* and used lignocellulosic biomass as raw material for furfural preparation. Furfural oxidation brought about the main product of SA and a small amount of maleic acid. Xylose was degraded to furfural and then oxidized to SA, while the reaction between acid-soluble lignin and SA intermediates inhibited the formation of SA. In addition, Xu et al. [87] proposed a co-fermentation method of SA and ethanol according to the characteristics of carbon source metabolism and product synthesis of *Saccharomyces cerevisiae* Y 2034 and succinic yeast ATCC 55618. By optimizing the culture conditions, nutritional components, and co-culture strategy, CO_2_ could be recycled during the fermentation of ethanol tail gas, and pentose and hexose in bagasse hydrolysate could be fully utilized. Succinic acid and ethanol with concentrations of 43.6 g/L and 40.5 g/L, respectively, could be obtained by the simple separation of fermentation products. This study provides a new insight into the bio-product processing from lignocellulosic biomass. Salma et al. [88] investigated the kinetics of SA production and the yield in the presence of sugar in the relevant synthesis medium by adding MgCO_3_ and FA as mineral carbon and SA precursor, respectively, to the medium of SA-producing actinobacteria. The results showed that the synthetic fermentation medium containing FA had the highest SA concentration and productivity of 0.49 mol L^−1^ and 0.48 g L^−1^ h^−1^, indicating that SA production could be increased by medium improvement. In addition, it was found that SA has an inhibitory effect on the growth of succinate synthase, so the SA yield can be improved by switching the process from batch mode to continuous mode during fermentation. This finding provides a valuable reference for research on the sustainable utilization of energy.

The regulation of key enzymes, development of genetic engineering strategies, and optimization of fermentation processes and media can be effective ways to further optimize the metabolic pathways of the strains and improve product production capacity while reducing the difficulty in by-product generation and product isolation. Compared with chemical synthesis, microbial fermentation using lignocellulosic biomass as a substrate for SA preparation boasted the advantages of low emission, low cost, renewable raw materials, and cost reduction of 50% [89]. After microbial fermentation, the fermentation broth containing organic acids must be separated and purified, and the downstream isolation process needs to be optimized to simplify the isolation and purification process as much as possible so as to reduce isolation costs and improve the recovery and purity of SA. Transgenic strains with customized redox potential balance can also be used to control the redox potential fermentation curve so as to determine the potential bottleneck of strain improvement and achieve the maximum output of various required metabolites [12]. This is an expensive process covering more than 50% of the total production cost because the broth contains many substances, such as bacteria, salt, protein, and other metabolites [90]. Therefore, it is necessary to develop an economical and environment-friendly separation and purification process to separate SA. Many downstream separation processes, including calcium precipitation, electrodialysis, direct crystallization by acidification or using cation exchange resin, salting out, and reactive extraction, have been applied for SA separation [91].

### Application prospect of succinic acid and its derivatives

**Fig. 8:**
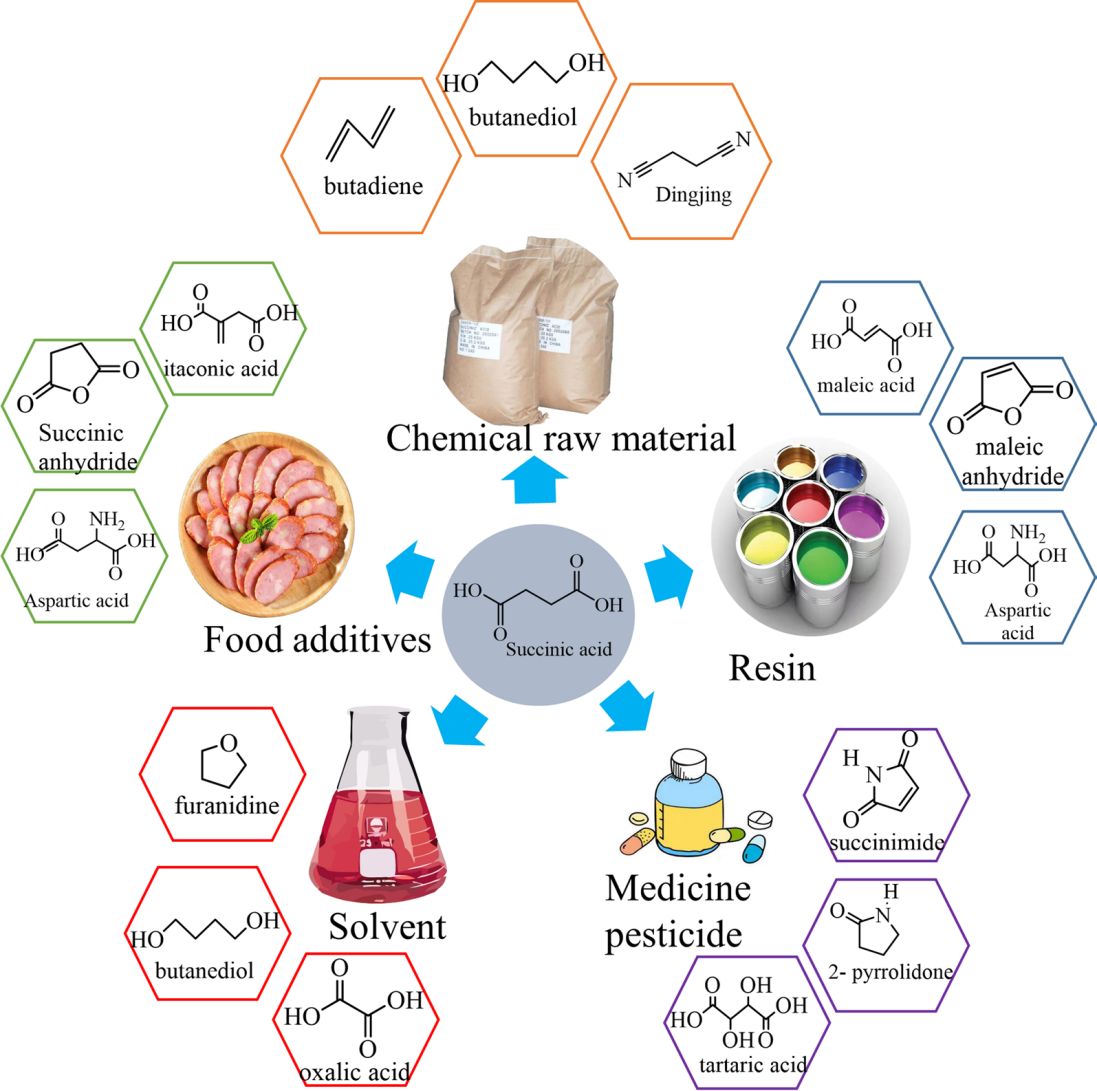
Utilization of succinic acid derivatives

Succinic acid is an important chemical material widely used in modern industry and has huge market demand. Several main chemical components can be produced by the chemical conversion of SA. At present, SA has been applied in the food industry, pharmaceutical industry, chemical industry, etc. Due to the existence of its functional groups, SA can be catalytically converted into various intermediates such as maleic anhydride, 1,4-butanediol, tetrahydrofuran, and γ-butyrolactone. Figure 8 shows the utilization of several important derivatives of SA. Succinic acid and its derivatives are mainly used as medicines, adhesives, solvents, and polymer synthesis intermediates. Additionally, they are important components of food additives, cosmetics, pharmaceuticals, biopolymers, solvents, plasticizers, and fine chemicals.

Due to the great potential of SA and its derivatives, they have been widely explored by researchers in recent years. Okur et al. [92] used SA as a matrix material to encapsulate model volatile vanillin, which can be retained for a longer period of time, as low molecular weight compounds are denser than high molecular weight compounds, which opens a new way to encapsulate volatiles in low molecular weight compounds. Succinate esters are promising environmentally friendly plasticizers for PVC that can replace toxic phthalate plasticizers. They are not only less toxic than phthalate plasticizers, but also have better tolerance to plasticizer migration from the polymer matrix. Elsiwi et al. [93] synthesized di-*n*-heptyl succinate from renewable raw materials and obtained a biodegradable and green PVC plasticizer. In addition, the production cost can be greatly reduced by optimizing the production process of SA derivatives. Chen et al. [94] improved the yield of SA dehydration to succinic anhydride to 80% by improving the catalyst, and this research has greatly contributed to the industrialization of SA.

In addition, succinate acts as a metabolic intermediate of the tricarboxylic acid cycle, is involved in energy supply and plays a role in metabolic reprogramming. As an epigenetic regulator, it is also involved in gene transcription, translation and post-translational modifications. Liu et al. [95] discussed the role of SA in the regulation of sepsis and demonstrated its effect on sepsis and its therapeutic potential. Janni et al. [96] found that the hemi-esters and hemi-amides of SA, sodium dodecyl succinate, sodium dodecyl succinate amide, and sodium cetyl succinate are useful surfactants for personal care formulations. This finding provides a valuable reference for the application of SA in pathology. In addition, SA-derived biodegradable polymers, succinic anhydride and polyamides indicate the huge application value and potential of SA [97]. Therefore, despite the long history and wide application of SA and its derivatives, they still have great development potential and deserve in-depth research in the direction of greener, more efficient, and lower-cost production.

## Conclusions

Succinic acid preparation by lignocellulose hydrolysis still has some shortcomings: the high cost and the long period of raw material pretreatment, the low cellulose hydrolysis rate, and the produced easy-to-decompose glucose. There are many by-products during acid production, increasing the difficulty of separation. The saccharification-hydrolysis of cellulose catalyzed by solid acid has become an efficient choice. The whole process of lignocellulosic biomass conversion to SA, from raw material pretreatment to cellulose saccharification and to glucose fermentation, involves catalytic hydrolysis with hydrolase addition. Therefore, to improve the SA yield, the characteristics, catalytic sites, and catalytic mechanism of hydrolase can be studied. Additionally, novel enzymes with high catalytic efficiency and excellent thermal stability are expected to be discovered, and economic and environment-friendly processes should be performed to achieve a higher yield and concentration of SA.

## Data Availability

Not applicable.

## Abbreviations

SA: Succinic acid
FA: Fumaric acid
TCA: Tricarboxylic acid cycle
DOE: Department of Energy
IEA: International Energy Agency
LHW: Liquid hot water
IL: Ionic liquid
*E. coli*: *Escherichia coli*
PEP: Phosphoenolpyruvate
PPC: Phosphoenolpyruvate carboxylase
PCK: Phosphoenolpyruvate carboxykinase
OOA: Oxaloacetate
